# Corrosion Behavior of AISI 316L Stainless Steel Used as Inner Lining of Bimetallic Pipe in a Seawater Environment

**DOI:** 10.3390/ma14061539

**Published:** 2021-03-21

**Authors:** Daquan Li, Qingjian Liu, Wenlong Wang, Lei Jin, Huaping Xiao

**Affiliations:** 1 China National Offshore Oil Corporation (CNOOC) China Ltd. (Zhanjiang), Zhanjiang 524057, China; lidc@cnooc.com.cn (D.L.); wenlong19832020@163.com (W.W.); 2 Tianjin Key Laboratory for Advanced Mechatronic System Design and Intelligent Control, School of Mechanical Engineering, Tianjin University of Technology, Tianjin 300384, China; 3 National Demonstration Center for Experimental Mechanical and Electrical Engineering Education (Tianjin University of Technology), Tianjin 300384, China; 4 China National Offshore Oil Corporation (CNOOC) (Tianjin) Pipeline Engineering Technology Co., Ltd., Tianjin 300461, China; jinlei135@163.com; 5 College of Mechanical and Transportation Engineering, China University of Petroleum-Beijing, Beijing 102249, China; hxiao@cup.edu.cn

**Keywords:** bimetallic pipes, corrosion cracking, seawater simulated solution, long-term corrosion tests

## Abstract

Seawater leakage commonly leads to corrosion in the inner lining of submarine bimetallic pipes, with significant financial implications for the offshore oil and gas production industry. This study aims to improve understanding of the performance of bimetallic pipes by investigating the corrosion behaviors of mechanically bonded 316L stainless steel. Immersion experiments were conducted in a seawater environment, under both atmospheric conditions and high temperature and high pressure conditions, and corroded surfaces were examined using scanning electron microscopy (SEM) and energy-dispersive X-ray spectroscopy (EDS) to reveal micromorphology and elementary compositions. The results demonstrated that the corrosion rates of the bonded 316L specimen were between 5% and 20% higher than those of specimens without bonding under atmospheric conditions. This is attributed to the stress cracking that occurs during corrosion. Under high temperature and high pressure conditions, the corrosion rates were remarkably increased (91% to 135%) and the corrosion process took longer to reach equilibrium. This may be attributed, firstly, to the products becoming increasingly porous and weak, and also to the fluid stress caused by stirring in these experiments to simulate seawater movement.

## 1. Introduction

Bimetallic pipes, designed to improve anti-corrosion performance in oil and gas production pipelines, provide a wide range of application prospects due to their excellent mechanical properties and capacity to resist corrosion [1,2,3]. They are produced by the mechanical or metallurgical bonding of ordinary, usually carbon steel, pipes with an inner lining of corrosion-resistant alloy. Stainless steel AISI 316L and AISI 304 are commonly used for this purpose. Compared to traditional anti-corrosion approaches, such as corrosion inhibitors, plastic inner coatings and other corrosion-resistant alloys, bimetallic pipes achieve a superior balance between anti-corrosion performance and cost. 

In the oil and gas production industry, however, the corrosion of offshore pipes is a significant and ongoing challenge [4,5]. Submarine pipes are commonly subjected to damage caused by the mechanical impacts of anchors and other heavy objects. This leads to cracks in the pipeline through which seawater leaks, introducing new challenges for the anti-corrosion performance of the inner lining of bimetallic pipes. The anti-corrosion capabilities of 316L and 304 are based on the formation of a passivation film created by the oxidation reaction or chemical adsorption of atoms or ions on the surface [6,7,8,9]. However, the seawater environment includes a high concentration of halide ions, notably chloride ions and bromide ions, which react easily with the passivation film and lead to decomposition [10,11], consequently exposing the substrate to surface pitting caused by electrochemical reactions [12,13,14]. Moreover, these chloride ions also hinder the formation of the steel’s passive film, thereby accelerating the corrosion process and, as the pitting gradually evolves and expands, deep pits or even corrosion perforations occur. In addition, as the lining layer is cold-worked during the mechanical bonding process, a certain amount of residual stress remains during its service period, further contributing to the challenge of bimetallic pipe stress cracking and corrosion [15,16,17]. Yoshitake et al. investigated the corrosion behaviors of centrifugally cast X52/625 and 825 bimetallic pipes and found the intergranular corrosion rate of the 825 to be significantly lower than that of the 625 [18]. Zhang et al. carried out corrosion tests on the X52/825 welding gap under both high temperature and high pressure conditions with CO_2_ and H_2_S [19], thereby demonstrating that the root welding gap provided excellent anti-corrosion and anti-stress cracking capabilities.

Long-distance oil and gas pipelines must withstand harsh environments with high temperatures and high pressure, which makes the design of bimetallic pipes complex. Although extensive investigations have reported on the corrosion of stainless steel, little research has been done on the corrosion behavior of this material when applied as the inner lining of a bimetallic pipe under complex conditions. In order to better understand the performance of bimetallic piping and predict its service life in offshore applications, it is necessary to explore the corrosion behavior of stainless steel with residual stress under the synergetic effects of seawater and extreme conditions (high temperature and high pressure). Thus, in this study, the corrosion behaviors of AISI 316L, with residual stress under high temperature and high pressure conditions in a seawater environment, are investigated.

## 2. Materials and Methods

An 18-inch bimetallic pipe with a 316L inner lining and an X65 carbon steel substrate, bonded using a mechanical approach, was used in this study to prepare the specimens of 316L. Specifically, a 316L pipe was put into an X65 pipe and then the two ends of the pipes were sealed. Hydraulic pressure was applied to the outer X65 pipe to produce plastic deformation until the two pipes were mechanically bonded together. The residual stress of the 316L layer was measured using a hole-drilling strain-gage method, according to the ASTM E837-01 standard [20], and was found to range from ~100 Mpa to ~140 Mpa. In addition to the 316L bonded with carbon steel, untreated 316L was also examined to provide data for comparative analysis. 

**Figure 1:**
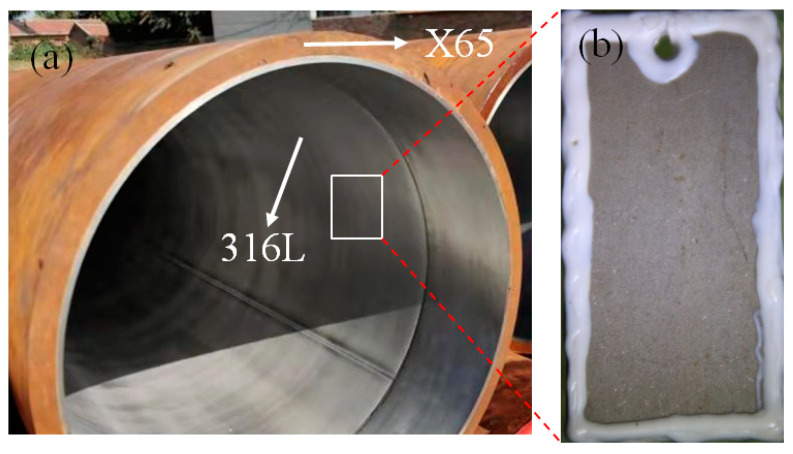
(**a**) Bimetallic pipe; (**b**) 316L specimen used in corrosion experiment.

The 316L samples, both with and without residual stress, were processed into specimens (60 mm × 30 mm × 3 mm) using the wire-cutting method (Figure 1). The specimens were ultrasonically cleaned using ethanol-acetone-ethanol to remove oil and other surface contaminants. Silicone was used to cover the backs, edges and holes of the specimens, thereby ensuring that only the front surfaces were exposed during the subsequent immersion experiments.

The corrosion behavior of the 316L specimens in the seawater environment was investigated through immersion experiments carried out in a hydrothermal reactor. To simulate the corrosion environment within an atmospheric condition, specimens were fully immersed in seawater collected from the Port of Tianjin in Bohai Bay, Tianjin, China. The reactor pressure and temperature were set to 0.8 MPa and 20 °C, respectively, and the immersion experiments were conducted over periods of 10, 20, 30, 60 and 90 days. Furthermore, to simulate the conditions in which oil and gas would be transported within the pipes, the immersion experiments were also carried out at a high temperature of 90 °C and high pressure of 8 MPa. The simulated liquid was prepared according to the formula presented in Table 1 and the simulated gas was composed of 4% CO_2_ and 96% N_2_. The simulated liquid was stirred continuously at a speed of 50 r/min.

After immersion, the specimens were removed and rinsed using a solution of hydrochloric acid and hexamethylenetetramine to remove any corrosive products on the surface. They were then dried and placed in a vacuum oven for later analysis and testing. The corrosion rate of each specimen was calculated using the following equation:

(1) $$R=\frac{8{.76\times 10}^{7}\times (M-M1)}{S\times T\times D}$$

where R is the corrosion rate (mm/year), M is the weight of the specimen before immersion experiment (g), M1 is the weight of the specimen after immersion experiment (g), S is the surface area of the specimen exposed to seawater (cm^2^), T is the immersion time (h), and D is the density of the specimen (kg/m^3^). 

The specimens were polished and etched in aqua regia to obtain metallographic photographs. The microstructures of the 316L specimen surfaces were investigated using a metallographic optical microscope (GX51, OLYMPUS, Tokyo, Japan) and a scanning electron microscope (SEM) (Phenom Pro-X, Thermo Fisher Scientific, Waltham, MA, USA. Variations of the elementary composition of the specimens after the immersion experiments were examined using energy dispersive spectroscopy (EDS, Thermo Fisher Scientific, Waltham, MA, USA).

## 3. Results and Discussion

### 3.1. Microstructure and Micromorphology of 316L before Immersion

**Figure 2:**
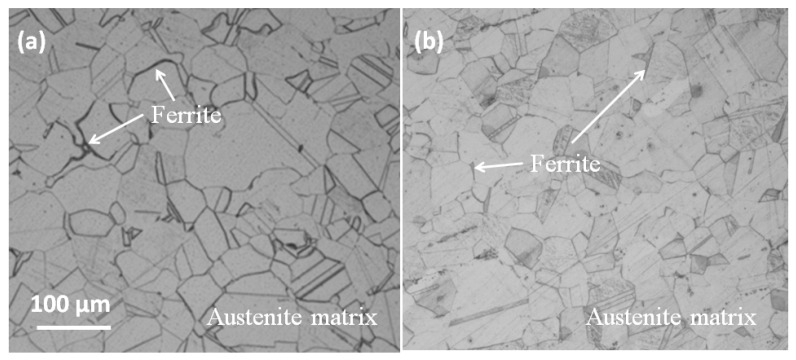
Microstructures of 316L (**a**) bonded with carbon steel and (**b**) without bonding.

Optical microstructure images of the 316L specimens, both bonded with carbon steel and without bonding, are presented in Figure 2. The images reveal the composition of 316L to include austenite grains and δ-ferrite with an average grain size of approximately 40 μm. No remarkable differences distinguish the two specimens (Figure 2a,b), which demonstrates that the mechanical bonding process had little impact on the microstructure of 316L, despite the significant level of residual stress generated during bonding.

**Figure 3:**
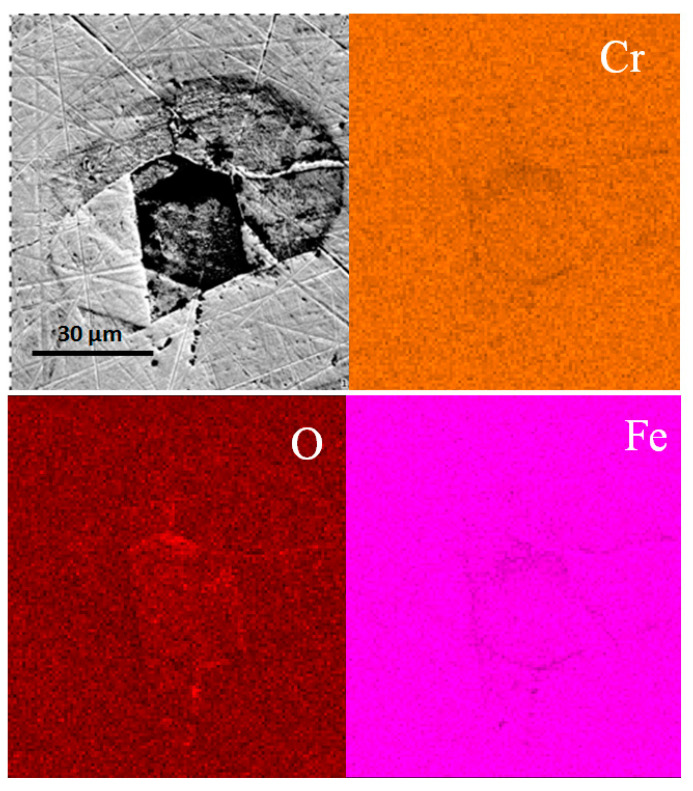
SEM morphologies and EDS mapping results of mechanically bonded 316L before immersion.

SEM morphologies and EDS mapping of the mechanically bonded 316L, presented in Figure 3, show a corrosion-sensitive area on its surface. The concentrations of carbon and oxygen are seen to be relatively higher in the center area in Figure 3, while iron elements are slightly reduced. The metallographic photograph in Figure 2 shows that ferrite is irregularly distributed on the surface and, as the solid solubility of C in the ferrite structure is low, this phenomenon is highly likely to promote corrosion. The ferrite-rich areas are, therefore, high-risk regions for corrosion. As seen in Figure 3, the corrosion product is iron oxide, the O concentration of which is higher and the Fe concentration lower than the matrix.

### 3.2. Corrosion Behavior under Atmospheric Condition

**Figure 4:**
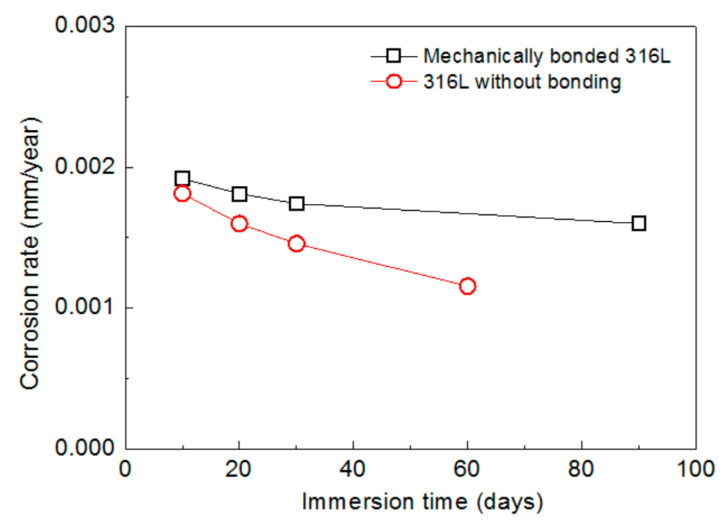
Variations in corrosion rates of 316L according to periods of immersion under atmospheric conditions.

The corrosion rates of 316L, measured following various periods of immersion under an atmospheric condition, are presented in Figure 4. The corrosion rate of the mechanically bonded 316L was found to be relatively higher than that of the 316L without bonding. This is mainly due to the significant level of residual stress produced during the bonding process. Compared to the unbonded specimen, the mechanically bonded 316L showed significantly higher susceptibility to stress corrosion cracking and, consequently, more severe corrosion. In addition, the corrosion rates of both types of 316L specimens reduced with immersion time, with the rate of the unbonded 316L reducing faster. By the 10th day of the immersion experiment, the corrosion rate of the bonded 316L was approximately 5% higher than that of its unbonded counterpart and, by the 30th day, this figure had increased to 20%, thus demonstraing that the impact of stress corrosion cracking increases the longer the product is immersed in this environment.

**Figure 5:**
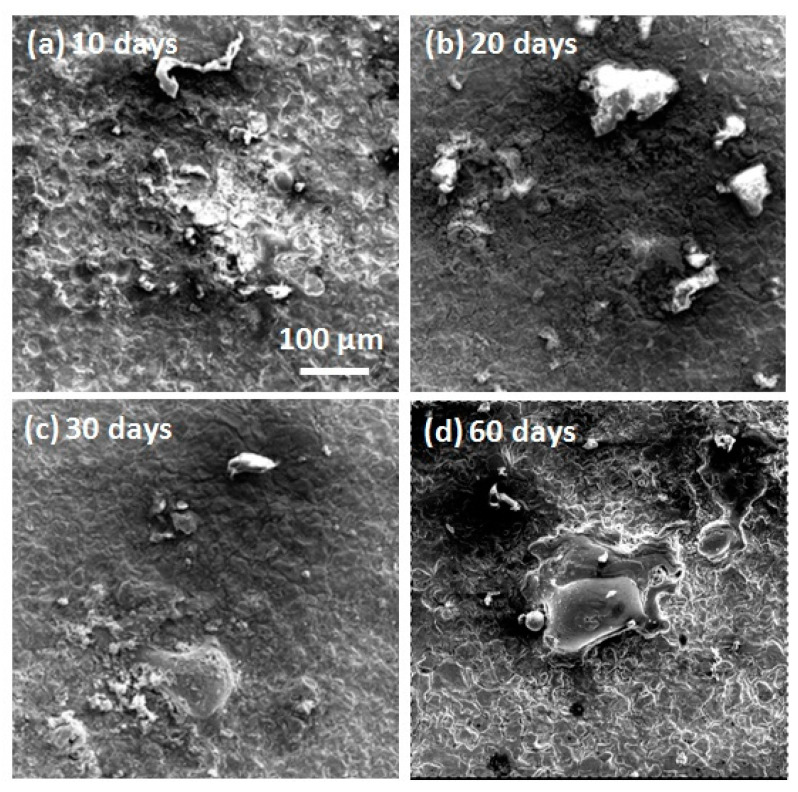
SEM morphologies of 316L over different periods of immersion. (**a**) after 10 days immersion; (**b**) after 20 days immersion; (**c**) after 30 days immersion; (**d**) after 60 days immersion.

**Figure 6:**
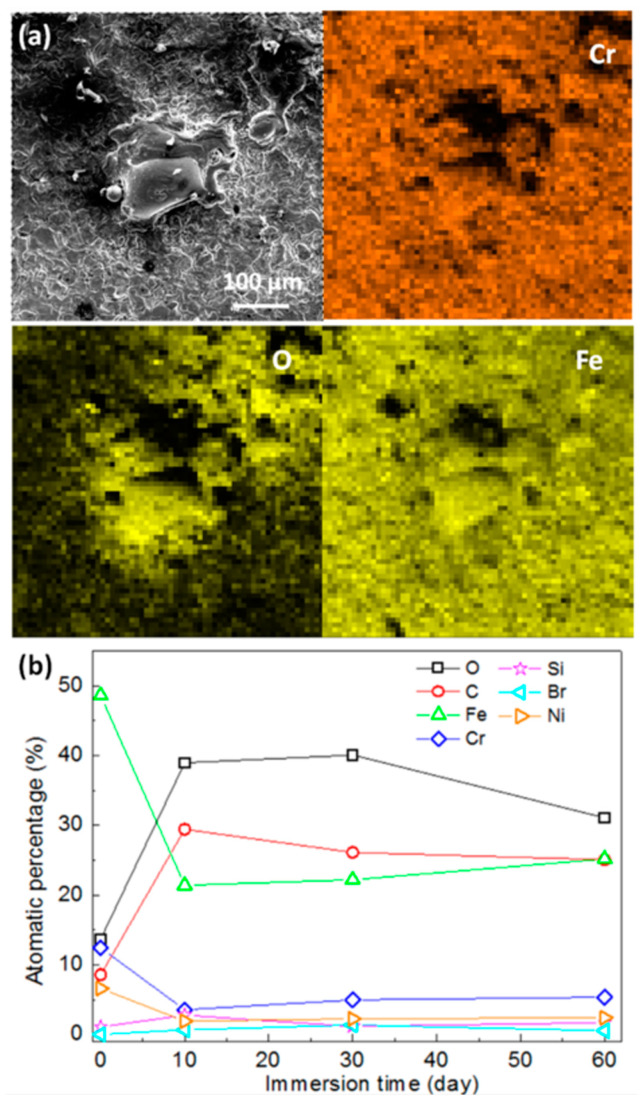
(**a**) EDS analysis of corroded 316L surface after 60 days’ immersion; (**b**) elementary composition of mechanically bonded 316L as a function of immersion time under atmospheric condition.

The micromorphologies of the mechanically bonded 316L specimen after different periods of immersion in seawater are shown in Figure 5, while the EDS mapping and elementary composition of the 316L specimens after immersion are presented in Figure 6. SEM characterizations of the corrosion products revealed them to be mainly loose protrusions on the surface. Combined with the EDS results in Figure 6, these corrosion products comprised mainly iron oxide with low concentrations of other elements, such as Cr, Ni, Mo, Si and Br. Variations in the elementary composition over the extended periods of immersion are presented in Figure 6b. 

Concentrations of Fe and Cr were found to decrease dramatically during the first 10 days of seawater immersion, indicating the rapid corrosion of the mechanically bonded 316L specimen. 316L austenitic stainless steel was designed to be highly resistant to corrosion, with relatively higher concentrations of Cr, Ni and Mo than carbon steel that contribute to the formation of a passivation film on its surface. However, in a seawater environment, Cl^−^ is actively contributing to the degradation of the passivation film and, consequently, the corrosion of 316L. Furthermore, stress cracking accelerates this process and hastens the rate of corrosion. In this study, the detected concentration of Br^−^ increased with immersion time, thus demonstrating an increased adsorption of these ions on the specimen surface. However, only a little Cl^−^ was detected on the corroded surface, indicating that this element remained mostly in an active state, continuously participating in corrosive reactions. In later stages of immersion, changes to the elementary composition of the specimen surface became less obvious, indicating that the corrosion process reaches an equilibrium.

### 3.3. Corrosion Behavior under High Pressure and High Temperature Conditions

**Figure 7:**
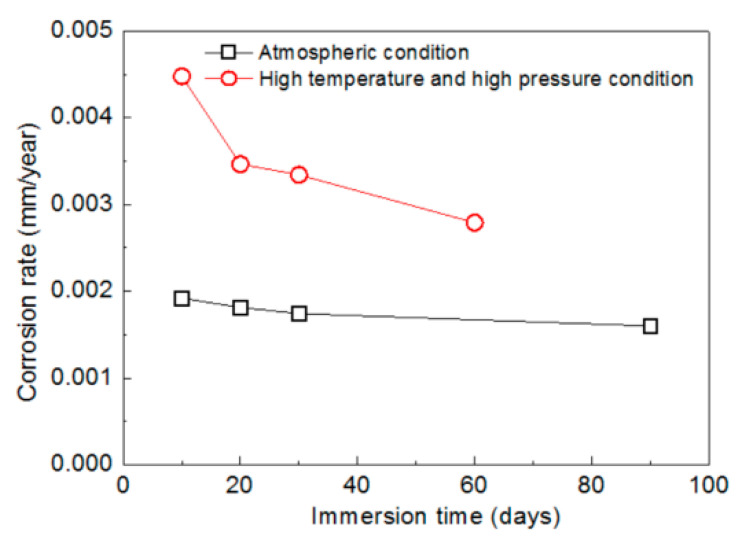
Variations in the corrosion rats of mechanically bonded 316L at different periods of immersion under an atmospheric condition and high temperature and high pressure conditions.

Comparative analyses of the corrosion rates of mechanically bonded 316L specimens under an atmospheric condition and a high temperature and high pressure conditions are presented in Figure 7. According to the measured results, the corrosion rate of the 316L stainless steel under the high temperature and high pressure conditions was significantly higher than that under atmospheric conditions. The corrosion rate after 10 days of immersion was calculated to be 0.0045 mm/year, which is more than twice that under the atmospheric conditions (0.0019 mm/year). After 30 days, the corrosion rate under the high temperature and high pressure conditions was still 92% higher than that under the atmospheric condition, thus demonstrating the significant acceleration of the corrosion reaction.

Under high temperature and high pressure conditions, the liquid was constantly stirred to create a liquid flow on the solid surface. This increased the flow of Cl^−^ on the 316L surface, thereby optimizing opportunities for reactions between the Cl^−^ and the stainless steel. In addition, the flow increased the shear stress of fluid on the surface of the material, damaging the protective film on the surface of the material. The damaged structures eventually tore off, leaving the metal matrix partially exposed [21,22] and, thus, accelerating pitting and increasing the rate of corrosion. 

**Figure 8:**
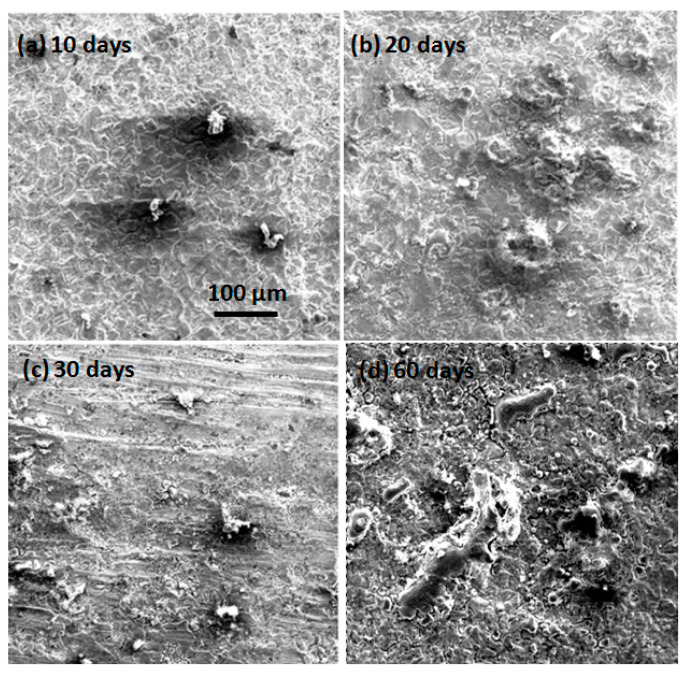
SEM morphologies of 316L immersed under high temperature and high pressure conditions. (**a**) after 10 days immersion; (**b**) after 20 days immersion; (**c**) after 30 days immersion; (**d**) after 60 days immersion.

**Figure 9:**
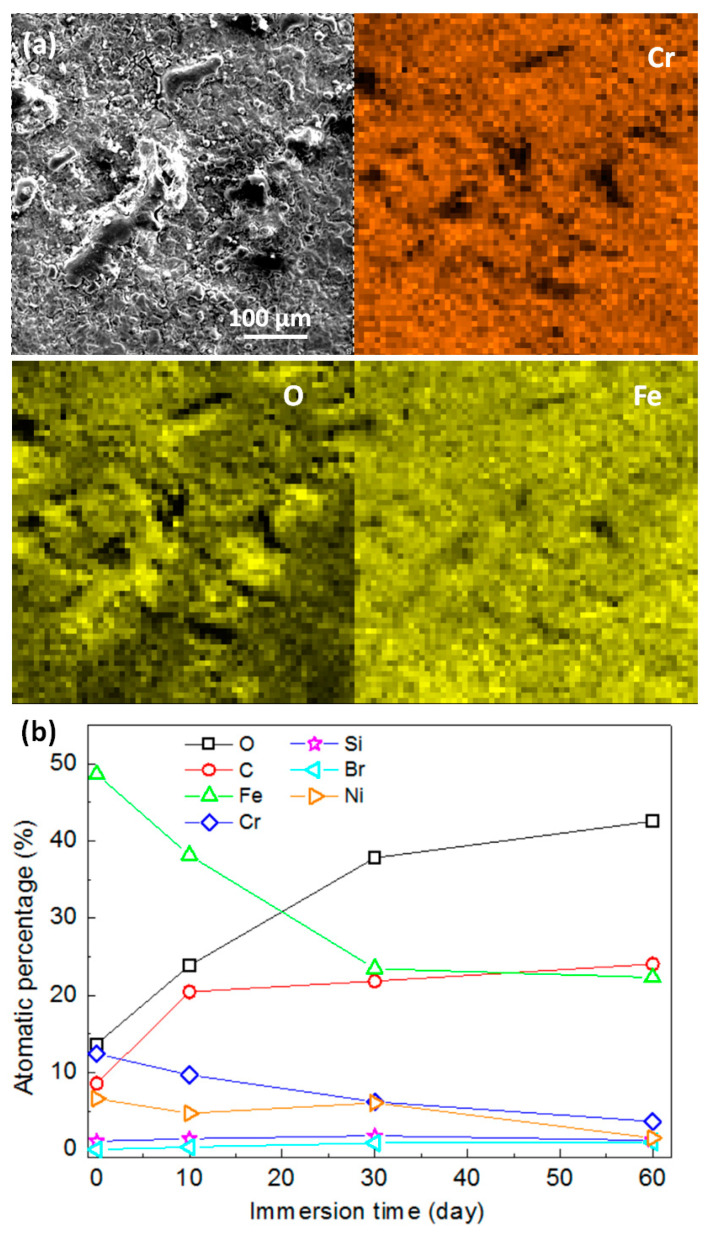
(**a**) EDS analysis of corroded 316L surface after 60 days’ immersion; (**b**) elementary composition of mechanically bonded 316L as a function of immersion time under high temperature and high pressure conditions.

The micromorphologies of the mechanically bonded 316L specimen after different periods of immersion under high temperature and high pressure conditions are presented in Figure 8. In the EDS mapping and elementary composition results, shown in Figure 9, it is clearly evident that the corrosion products under high temperature and high pressure conditions have a porous, loose structure. Compared to the corrosion products under the atmospheric conditions (Figure 5), those in Figure 8 appear more porous. Moreover, under a higher temperature with CO_2_, a non-protective and porous FeCO_3_ and Cr-O hydrate corrosion product film formed on the surface of the 316L. These corrosion products have low mechanical strength and are easily removed, leading to a high rate of corrosion [23,24]. Here, the structure became increasingly loose and porous the longer the specimen was immersed.

According to Waard et al. [25], Davies et al. [26] and Linter et al. [27], possible anode reactions during the corrosion process are:

Fe+OH^−^→FeOH+e (2)

FeOH→FeOH^+^+e (3)

FeOH^+^→Fe^2+^+OH^-^ (4)

Fe→Fe^2+^+2e (5)

Fe+H_2_O→Fe(OH)_2_+2H^+^+2e (6)

Fe+ HCO_3_^−^→FeCO_3_+H^+^+2e (7)

However, the corrosion mechanism of steel with CO_2_ is highly complex and affected by many factors, including temperature, pH value, CO_2_ partial pressure, medium composition, flow rate and flow state, and material surface conditions. The intermediate products created by CO_2_ corrosion vary according to changes in the experimental conditions and, therefore, the actual corrosion reaction process in this study could not be fully determined. A corrosion product with a porous structure was, however, observed in this study which is similar to the previously reported porous FeCO_3_. Variations in the elementary composition at different immersion times are presented in Figure 9b, from which it is obvious that the concentrations of Fe and Cr decreased quickly, while those of C and O increased in the first 30 days of immersion. Further changes in the concentrations of these elements became subsequently less remarkable and the composition of the corroded surface is believed to have become relatively stable. This suggests that, under a high temperature and high pressure condition, a longer period of time is required for the corrosion process to reach equilibrium. 

## 4. Conclusions

316L austenite stainless steel samples, mechanically bonded with carbon steel, were obtained from an 18-inch bimetallic pipe. The corrosion behaviors of this material were investigated in a seawater environment under both an atmospheric condition and high temperature and high pressure conditions. The experimental results show, firstly, that compared to the unbonded 316L specimens, the corrosion rate of the bonded 316L was 5% to 20% higher under the atmospheric condition, which attributable to stress corrosion cracking. Secondly, based on the elementary composition, the corrosion system reached equilibrium in approximately 10 days under the atmospheric condition. Lastly, the corrosion rate was elevated to between 91% and 135% under the high temperature and high pressure condition, and it took approximately 30 days for the corrosion system to reach equilibrium.

## Figures and Tables

**Table 1:** Formula for the simulated liquid used in high temperature and high pressure immersion experiment.

Ingredients	Concentrations/(g/20 L Ultrapure Water)
FeCl_2_·4H_2_O	0.0007
Mg(NO_3_)_2_·6H_2_O	0.1899
Ba(NO_3_)_2_	0.078
NaNO_3_	7.8359
Ca(NO_3_)_2_·4H_2_O	0.3067
NaS·9H_2_O	0.0022
CaCl_2_·2H_2_O	0.1654
KHCO_3_	0.4097

## Data Availability

The data presented in this study are available on request from the corresponding author. The data are not publicly available due to privacy restrictions.
